# Psychosocial impacts of a mouse plague and ongoing psychological stress

**DOI:** 10.1038/s41598-026-39861-1

**Published:** 2026-02-11

**Authors:** Aditi Mankad, Kerry Collins, Walter Okello, Lucy Carter, Peter R. Brown

**Affiliations:** 1 CSIRO Environment, GPO Box 2583, Brisbane, QLD 4101 Australia; 2 CSIRO Environment, GPO Box 1700, Canberra, ACT 2601 Australia; 3 CSIRO Health & Biosecurity, GPO Box 1700, Canberra, ACT 2601 Australia

**Keywords:** Biosecurity, Pest incursion, Public attitudes, Australia, Social science, Farming, Psychology, Natural hazards

## Abstract

In 2020-21, parts of eastern Australia experienced a severe mouse plague, estimated to have caused up to AUD$1 billion worth of damage. Yet, the mental health impacts of such a pest plague are not empirically known. The present study sought to quantify and model the psychosocial impacts of the 2021 mouse plague and measure ongoing psychological stress two years post-plague, to provide insights into social impacts and mental wellbeing during a significant biosecurity incursion. An online survey (*N* = 1,691) measured key psychosocial impact metrics included psychological traits, emotional states (depression, disgust, embarrassment), perceptions of social impacts (response costs, attitudes), support (social, government), and perceived threat of future plague. A partially mediated path model accounted for 74% of variability observed in perceived *severity of impacts* and *ongoing psychological stress. Severity of impacts* was predominantly influenced by perceived *response costs*, negative mood states (i.e. *depression*) and *disgust* elicited by the odour of mice. The most influential direct predictors of *ongoing psychological stress* were depressive symptoms at the time of the plague, and concerns that a future mouse plague would be dangerous or catastrophic to oneself. This study provides empirical insights into both acute social impacts during a significant pest incursion, as well as ongoing psychological stress. Social impacts are rarely measured or modelled; yet these factors are critically important in understanding the effect of severe pest incursions on communities whose livelihoods often depend on the agricultural sector.

**Supplementary Information:**

The online version contains supplementary material available at 10.1038/s41598-026-39861-1.

## Introduction

Mouse plagues occur somewhere in Australia every 3–4 years and once every 5–7 years in any particular region, causing substantial economic, social and environmental damage^1^. They are defined as synchronous irruptions, with populations exceeding 800 mice/ha over thousands of square kilometres^1^. Mouse plagues normally follow above average rainfall following 1–2 years of drought conditions, though rainfall by itself is not the main driver of mouse plagues^2–5^. Notable mouse plagues have occurred in New South Wales (NSW) in 1980 and 1984^1^, and again in 2010/11 following the Millenium Drought^6^. From mid-2020 until the end of 2021, a significant mouse plague affected areas of southern Queensland, western Victoria, South Australia and throughout regional NSW, with the latter region most severely affected^7^. Regular mouse monitoring coordinated by Australia’s national science agency, showed that mouse numbers increased substantially from low levels of activity in August and November 2020 to very high levels by March 2021 through central and northern parts of NSW^7^. Mouse numbers remained high until August 2021 and crashed by October 2021. [As the mouse plague reached its peak in 2021, we refer to it within text as the 2021 mouse plague, hereafter].

The present study seeks to understand, articulate, and quantify the psychological impacts of this 2021 mouse plague on communities in regional NSW. Previous research into the impacts of the 1993 mouse plague – the last significant mouse plague in Australia with recorded impacts – collected primarily economic data, with some presentation of social impacts in the context of broader economic losses^8^. However, there is almost no empirical literature in the biosecurity domain that examines the equally important psychological and social impacts affecting people after a plague or any other significant pest incursion. Knowing how communities are affected, and the psychosocial factors that place individuals most at risk, allows for the prevention and/or early intervention of severe social impacts.

### Current knowledge of mouse plague impacts

Mouse plague impacts are felt across all sectors, from producers, businesses, households, and individuals; on farms and in rural townships; in paddocks and sheds and inside people’s homes. Typically, economic impacts involve significant production losses in grain, grazing, fruit and vegetable crops, and intensive livestock industries, as well as the destruction of stored goods, machinery, equipment, infrastructure, houses, and personal possessions^8–10^. The direct economic impact on growers and the community of the 1993/94 mouse plague that affected the Australian states of Victoria and South Australia, for example, was conservatively estimated at more than $64.5 million^8^. The more recent 2011 mouse plague, which spread across four Australian States (Queensland, New South Wales, Victoria, and South Australia), reportedly caused over AUD $200 million in crop damage alone, affecting around 3 million hectares of crops^11,12^. The NSW Farmers Association (a farmer advocacy group) predicted during the 2020/21 mouse plague that it may cause losses of more than AUD $1 billion off the farmgate value of the NSW winter crop^13^.

However, the impacts of mouse plagues, particularly on households, cannot only be considered financially^14^. Mouse plagues have a significant social impact, and these are less articulated and quantified in the literature. The physical human health impacts are somewhat easier to measure and include increases in cases of rodenticide exposure, zoonotic disease transmission (e.g., leptospirosis) and exposure to food-borne bacterial and protozoal diseases (e.g., salmonellosis, cryptosporidiosis)^15^.

### Psychosocial impacts

Mice saturate both the farm and non-farm environments, infesting homes and causing damage to property inside and outside the home^16^. Mice do not just affect material or consumable goods, but they also likely cause psychological effects such as fear, panic or anxiety^7,17^and during a plague they can maintain a consistent presence in high numbers over the course of many months. Their lack of confinement and respect for ‘traditional’ pest limits, makes mice an overwhelming pest. When mice invade people’s homes they spoil food, clothing and furnishings, damage appliances and vehicles, and leave an odour of urine, faeces and decay that can linger long after the plague ends. Mice coming in physical contact with people, running over or biting them as they sleep, adds further stress^8,14^. Behavioural changes (e.g., risky use of poisons, uncharacteristic or remorseless treatment of mice), business closures, suicides and marital breakups have all been variously recognised as impacts of a mouse plague^8^.

#### Trauma

Experiencing a mouse plague can involve a subjective experience of trauma both in terms of having exposure to mice and the significant negative impact a plague has on one’s livelihood. *Trauma* can be defined as a threatening or harmful experience that causes an intense physical and psychological stress reaction with lasting adverse effect on one’s wellbeing^18,19^. It is noted that the American Psychiatric Association^20^ uses a much narrower clinical definition of trauma in their Diagnostic and Statistical Manual-5 (DSM-5), where trauma is clinically defined as in response to “*actual or threatened death*, *serious injury*,* or sexual violence*” (p.271).

Grey literature during the 2021 mouse plague offered insights into the likely mental health effects of coping with the psychological impact of mouse plagues, such as stress, anxiety, and fear^21–24^. Hodgins^17^ likened the psychological experience of a mouse plague to that of other disaster events such as drought and bushfire, particularly in the way that the mouse plague was not an acute event, and its effects were ongoing for an extended period. Hodgins^17^speculated that although much of the population would likely develop some form of resilience and cope with the effects, some people might be significantly affected after such a disaster.

To date there is scant literature testing these trauma hypotheses after a significant pest invasion amongst agricultural communities, and certainly no psychosocial investigations on the mental toll of mouse plagues. This demonstrates a significant gap in the biosecurity and pest science literature. A goal of the current research is to articulate communities’ experiences during a significant pest invasion and the potential long-lasting effects, and to consider whether the clinical definition of trauma should be extended to capture psychological impacts from these types of physically and emotionally threatening biosecurity incursions.

#### Post-traumatic stress

The American Psychiatric Association’s Diagnostic and Statistical Manual-5 (DSM-5)^20^ defines post-traumatic stress disorder (PTSD) as meeting four key criteria based on exposure to a traumatic stressor: (a) intrusive thoughts (e.g. flashbacks, dreams); (b) avoidance of stimuli associated with the event (e.g. places, people, memories); (c) negative cognitions and mood associated with the event (e.g. negative beliefs, depressive thoughts); and (d) increased arousal beginning or worsening after the event (e.g. startled responses, sleep disturbance, irritable behaviour). The DSM-5 considers PTSD to fall within the “Trauma and Stressor-related Disorders”, and we argue that while the mouse plague event may not strictly classify as clinical trauma, the mouse plague is a severe and adverse *stressor* that can elicit PTSD symptomology. Thus, we have used metrics to measure PTSD symptomology while clearly stating that our goal is not to *diagnose* PTSD but rather to describe individuals’ experiences of ongoing psychological stress post-plague. The current study argues that the 2021 NSW mouse plague was a significant traumatic event or stressor for the regional communities affected; and that, two years on, some people may still be experiencing measurable emotional disturbance and post-traumatic stress symptoms associated with the plague.

While agricultural disasters (e.g. pest plagues) have not gained much attention in the trauma literature, there is quite a bit of published research examining psychological impacts after significant climate disasters such as bushfires (see^25^ for a systematic review). Literature in the bushfire context consistently shows that while a majority of people were able to cope with these disasters over time and manage their level of psychological stress, a small but significant proportion of the affected population (~ 15%) do not cope well and continue to be impacted up to 4 years post-bushfire, and for up to 7 years post-bushfire amongst firefighters^25,26^.

Interestingly, personality research has found that among all personality traits, *neuroticism* has the potential to both increase individual vulnerability to PTSD and magnify PTSD symptom severity. For example, the climate disaster literature has found that individuals with higher levels of catastrophising after a natural disaster tended to score higher on neuroticism, and both neuroticism and catastrophising were found to be major risk factors for PTSD symptoms post disaster^27,28^. Therefore, it is reasonable to hypothesise that individuals higher in neuroticism may experience more severe impacts from a mouse plague and that neuroticism may also influence individuals’ ongoing psychological stress post-plague.

#### Past experiences and social support

The climate disaster literature has found that previous exposure to a disaster (e.g. drought, bushfire) and an acceptance of inherent geographical risk or regular seasonal threats of a disaster event can contribute significantly to post-disaster stress reduction^28–31^. That is, if people believed drought was a ‘normal’ event, given biophysical characteristics of where their property was situated, the perception of normality might mitigate the stress response. Further, those who have experienced previous mouse plagues are usually better prepared and better able to cope compared to those who have not^31^.

Other literature shows that amongst farmers and communities experiencing drought-related stress, individual impacts of stress were intertwined with the impacts of drought on oneself, their families, and their community^32^. That is, social impacts from disaster events were perceived and evaluated collectively, rather than individualistically. This more collectivist orientation when experiencing a disaster, where the community’s needs are considered alongside one’s own, can foster strong social support networks^33^; and, there is evidence to suggest that increased social support can protect against negative mood states (e.g. depression, anxiety) and PTSD^34–36^. Thus, it is possible that social support may be a protective factor in an individual’s experience of mouse plague impacts and should be considered when examining impacts and ongoing psychological stress. It should be noted, however, that while collectivist orientations are associated with more supportive social interactions within an ingroup, some research suggests individuals within that group may be more hesitant to seek personal support to avoid burdening others^37^. Indeed, Caughley and colleagues^8^found that during the 1993/94 mouse plague, some people internalised their stress and were reluctant to discuss their problems with others, and as such the community did not draw together as it would typically when facing a natural disaster. These inconsistent findings are interesting and warrant further investigation to better understand the role that social support might play during a pest incursion.

### Present study

This study sought to quantify and model the psychosocial impacts of the 2021 mouse plague on affected communities, and measure ongoing psychological stress two years post-plague, to provide insights into psychosocial impacts during a significant biosecurity incursion. It was hypothesised that key variables such as emotions, attitudes, social support and response costs would influence perceived social impacts, and that those factors along with social impacts would in turn influence one’s level of ongoing psychological stress after the event. Due to the limited empirical literature on social impacts of pest invasions, key variables were included based on past disaster literature; anticipated relationships between the specific factors were largely exploratory.

## Methods

### Participants and procedure

**Fig. 1:**
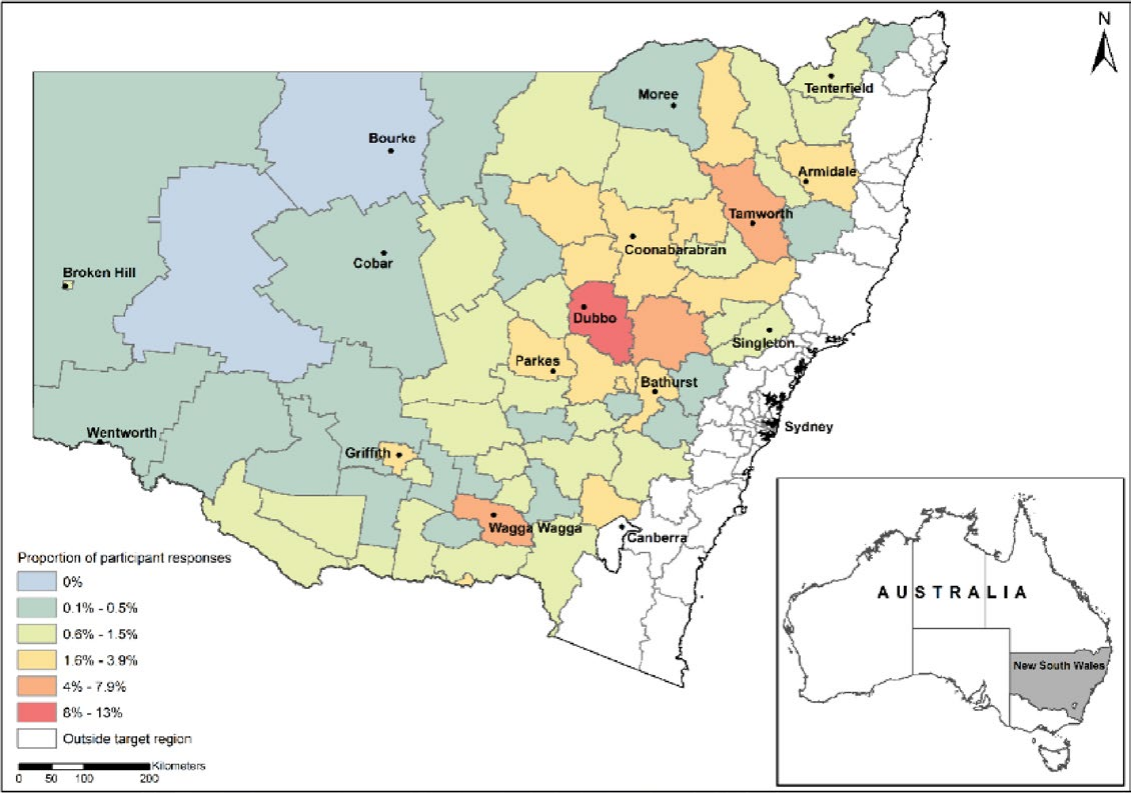
Distribution of participants across regional New South Wales, which reflects the distribution of mouse control rebates across the region, from July 2021-January 2022.

A total sample of 1,691 residents in regional New South Wales (NSW), Australia, participated in this study (28% male, 71% female, 0.3% non-binary and 0.7% prefer not to say), with an average age of 48.70 years (SD = 15.64 years). Participants were recruited for the online survey via two pathways: (1) online research panel, matching age and gender with Australian Bureau of Statistics representativeness for the target regions (*n* = 806)^38^; and, (2) distribution of a survey link via industry newsletters and social media (*n* = 885). Eligible participants included those from areas where the NSW government saw the highest distribution of mouse control rebates during the 2021 mouse plague (Fig. 1). Participants were required to be ≥ 18 years and self-report that they were affected by the mouse plague in some way (e.g. mice affected their household or business).

**Fig. 2:**
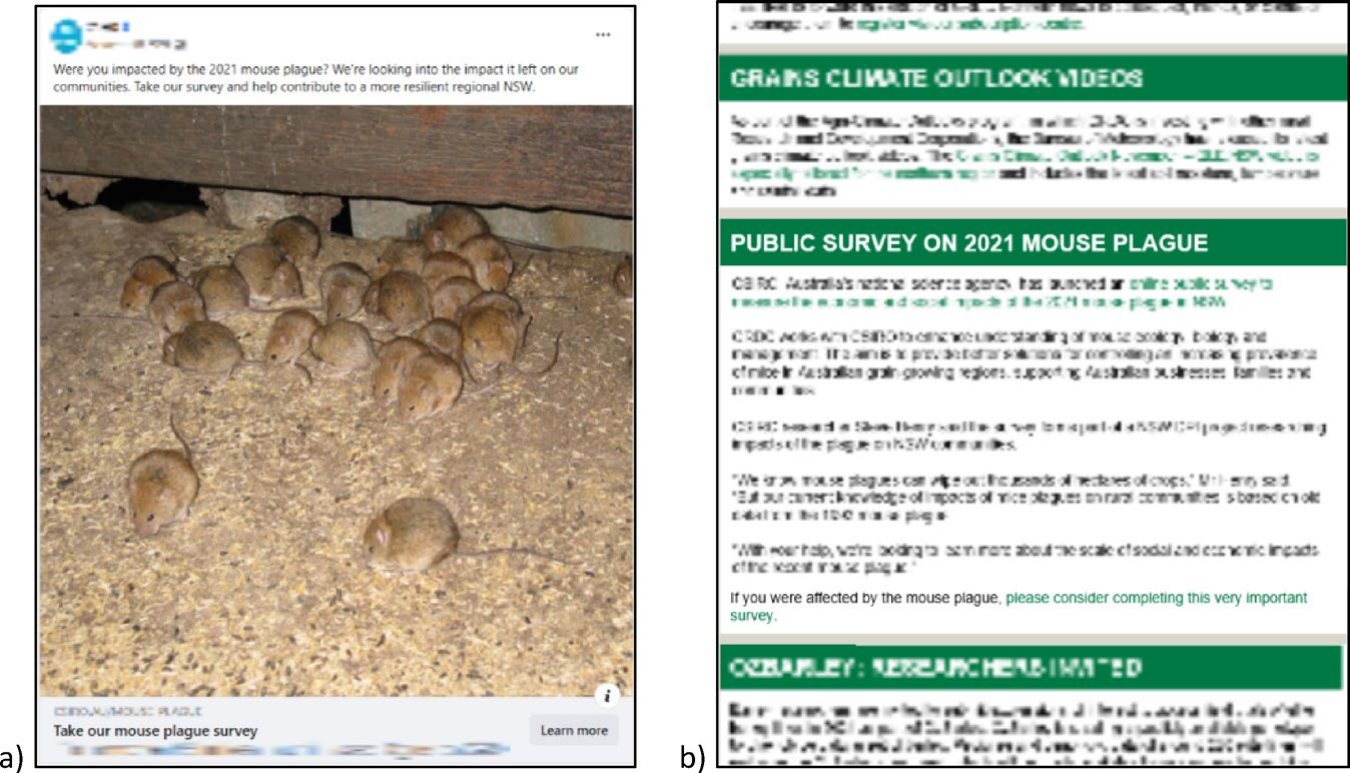
Example images of survey recruitment pathways via (**a**) Social media, and (**b**) industry newsletter. [Source: (**a**) Facebook™; (**b**) Grains Research & Development Corporation].

The survey was hosted by an accredited online research Provider [Accredited member of the Australian Data and Insights Association, ISO 20252 ‘Market, opinion, and social research’ (ISO 20252:2019) status^39^, who carried out the representative panel recruitment process between October-December 2023 (approximately two years after the NSW plague officially ended). Panel members in the target region were emailed an invitation to participate in the survey and received a token incentive (e.g. redeemable membership points) for completion; this process was managed by the online research Provider, independent of the research team. The Provider also created a direct survey link for distributing through industry channels (e.g. Grains Research and Development Corporation newsletter) and via social media, where the link was shared using two unique paid advertisements on Facebook™ (Fig. 2). Participants took an average of 14 min (panel recruitment) and 17 min (social media link) to complete the survey. The survey response rate was calculated as approximately 10% for the research panel participants, with a survey dropout rate of 13%; a 10% completion rate was calculated for the survey distributed via external link.

This research was approved by an accredited Human Research Ethics Committee (Ref: 179/22), in line with Australia’s National Statement on Ethical Conduct in Human Research (https://www.nhmrc.gov.au/about-us/publications/national-statement-ethical-conduct-human-research-2025).

### Measures

Development of the survey instrument was guided by relevant literature, as well as an initial qualitative investigation of regional NSW residents who experienced the mouse plague and shared their experiences via a series of regional focus group discussions^40^.

#### Demographic and descriptor variables

General demographic indices were included in the survey: *age* (open numerical response); *gender* (categories: male, female, binary, prefer not to answer); and self-categorisation as a *farmer* or non-farmer, to enable subsequent comparisons across variables of interest. Other general descriptors were also included to provide more detail and situational context regarding the sample (e.g. years lived in local area, past experiences with a plague); a presentation of these can be found in Supplementary Table S1.

#### Independent (predictor) variables

Several independent variables were included to adequately capture the varied social impacts on individuals in retrospect and over time (Table 1). The variables were organised conceptually:

- Psychological traits (neuroticism);
- Emotional states at the time of the plague (depression, disgust, embarrassment);
- Psychological perceptions of social impacts of the plague (attitudes, response costs, perceived control, animal welfare, poor behaviours);
- Support received during the plague (social support, authority support, role of government);
- Future-oriented perceptions (threat vulnerability, threat severity, future preparedness).

Generally, higher scores on each variable indicated stronger affiliation with the characteristic described. Given the applied nature of this research, some variables in the survey were measured using a single item for efficiency. These items held high face and content validity, and a growing body of literature suggests that single item measures can be useful predictors for measuring cognitive and affective outcomes in applied research^41,42^.

**Table 1:** Survey items measuring severity of impacts and ongoing psychological stress from the 2021 NSW mouse plague, covering psychological traits, emotional states, impact-oriented perceptions, and future threat perceptions.

Variable	Example items	Response scale
Neuroticism Cronbach’s α = 0.88 Adapted from [43], [44], [45]	*I see myself as someone who…* - *Is depressed*,* blue* - *Is relaxed*,* handles stress well* - *Can be tense* - *Worries a lot* - *Is emotionally stable*,* not easily upset* - *Can be moody* - *Remains calm in tense situations* - *Gets nervous easily*	1 = strongly disagree 3 = neither agree or disagree 5 = strongly agree
Depression Cronbach’s α = 0.94 Adapted from Patient Reported Outcomes Measurement Information System^®^ (PROMIS^®^) Short Form v1.0 - Depression 8b [46], [47], [48]	*Thinking back to how you were feeling during the 2021 mouse plague*,* please rate how often you felt any of the following*: - *I felt worthless* - *I felt that I had nothing to look forward to* - *I felt helpless* - *I felt sad* - *I felt like a failure* - *I felt depressed* - *I felt unhappy* - *I felt hopeless*	1 = never 2 = rarely 3 = sometimes 4 = often 5 = always
Disgust-smell	*To what extent did the smell of mice bother you during the 2021 mouse plague*	1 = not at all bothered 3 = moderately bothered 5 = extremely bothered
Embarrassment	*I felt embarrassed about having mice in my home or business*	1 = strongly disagree 3 = neither agree or disagree 5 = strongly agree
Attitudes Cronbach’s α = 0.74 Adapted from [49], [50], [51], [52], [53]	*I felt that my personal actions in dealing with the mouse plague were…* *…ineffective – effective* *…insufficient – sufficient* *…risky – safe* *…foolish – wise* *…disorganised – organized* *…unethical – ethical*	5-point polar adjectives scale For example: 1 = risky .……………… 5 = safe 1 = foolish ……………0.5 = wise
Response costs Cronbach’s α = 0.89	*Controlling mice during the 2021 mouse plague …* … *required* a lot of effort *from me* *… took up a lot of my* time … *cost me a lot of* money	1 = strongly disagree 3 = neither agree or disagree 5 = strongly agree
Perceived control	*I felt as though I was in control of managing mice on my property during the 2021 mouse plague*	1 = strongly disagree 3 = neither agree or disagree 5 = strongly agree
Animal welfare Cronbach’s α = 0.76	*During the mouse plague…* - *The welfare of mice was a major concern for me* - *I felt sad killing the mice* - *Most people in my community felt bad killing mice* - *I felt that the mice should not be disturbed*	1 = strongly disagree 3 = neither agree or disagree 5 = strongly agree
Poor behaviours	*The impacts of the mouse plague were so bad that I did things at the time that I knew were wrong (e.g. knowingly using the wrong bait)*	1 = strongly disagree 3 = neither agree or disagree 5 = strongly agree
Social support Cronbach α = 0.57 Adapted from the Oslo Social Support Scale – OSSS-3 [54]	*During the mouse plague…* *…how many people could you count on to help you?*	1 = none 2 = 1–2 people 3 = 3–5 people 4 = 5+ people
	*…how much interest and concern did people show for you?*	1 = none 2 = little 3 = uncertain 4 = some 5 = a lot
	*…how easy was it to get practical help from neighbours if you needed it?*	1 = very difficult 2 = difficult 3 = possible 4 = easy 5 = very easy
Communication from authorities	*I believe the X Government were proactive in informing me about the 2021 mouse plague*	1 = strongly disagree 3 = neither agree or disagree 5 = strongly agree
Authority support	*I was satisfied with the level of support provided to me by the X Government during the 2021 mouse plague*	1 = strongly disagree 3 = neither agree or disagree 5 = strongly agree
Role of government	*I believe the role of the X Government during the 2021 mouse plague was to …*	Open text response
Threat vulnerability	*It is likely that I will get mice on my property if another mouse plague happens*	1 = strongly disagree 3 = neither agree or disagree 5 = strongly agree
Threat severity	*I am worried that a future mouse plague will be dangerous or catastrophic to me*	1 = strongly disagree 3 = neither agree or disagree 5 = strongly agree

#### Dependent (mediating) variable: severity of impacts

The four items comprising *severity of impacts* were specifically developed for this survey based on literature^8,40^and were designed to operationalise individual perceptions of both personal and community impacts (Table 2). Past qualitative research in the context of disaster events highlights the reality of both individual and ‘collective’ trauma, perhaps more so amongst those in agricultural and rural communities, where external support resources can be difficult to access and individuals within these communities rely on each other for reference^55,56^. The four items were combined into a single summed score (range: 4–20) after a reliability test confirmed that the items reflected good internal consistency (Cronbach’s *α* = 0.85); higher scores indicated greater severity of impacts.

**Table 2:** Four items and their response scales, measuring the severity of impacts dependent variable.

Items	Response scale
The impact of the 2021 mouse plague on my community was…	1 = not severe 3 = moderately severe 5 = extremely severe
The impact of the 2021 mouse plague on me was…	1 = not severe 3 = moderately severe 5 = extremely severe
Thinking of all the stressful events that you have experienced in your life so far, how does the 2021 mouse plague compare in terms of stress severity?	1 = not stressful at all 3 = moderately stressful 5 = the most stressful event
Thinking of all the significant negative events that your community has experienced in the last few years, how does the 2021 mouse plague compare in terms of stress severity?	1 = not stressful at all 3 = moderately stressful 5 = the most stressful event

#### Dependent (outcome) variable: ongoing psychological stress

*Ongoing psychological stress* measured current experiences of stress (i.e. approximately two years after the 2021 mouse plague). The variable was measured using an adapted form of the Impact of Events Scale-Revised (IES-R)^57,58^ which measures three of the four main criteria for post-traumatic stress disorder (PTSD) as defined in the DSM-5^20^: *avoidance*, *intrusion*, and *hyperarousal* (Table 3; Cronbach’s α = 0.94). It is important to note that the use of IES-R in this study was to assess the symptoms of PTSD, and *not* as a diagnostic instrument. While Beck and colleagues^59^cite evidence suggesting the IES-R can discriminate between individuals with and without PTSD, further evidence of its utility as a diagnostic tool remains limited. Thus, the decision to refer individuals for diagnosis based on the IES-R scores alone is unlikely^58,59^.

**Table 3:** A measure of ongoing psychological stress using an adapted form of the impact of events Scale-Revised^57,58^; example items are provided, derived from the full 22-item scale.

Subscale	Items	Response scale
Intrusion *(7 items)* Cronbach’s α = 0.89	- *Any reminder brings back feelings about the mouse plague* - *Other things keep making me think about the mouse plague* - *I think about the mouse plague when I don’t mean to*	1 = Not at all 2 = A little bit 3 = Moderately 4 = Quite a bit 5 = Extremely
Avoidance *(8 items)* Cronbach’s α = 0.85	- *I stay away from reminders of the mouse plague* - *I try not to think about the mouse plague* - *I am aware that I still have a lot of feelings about the mouse plague*,* but I don’t deal with them*	
Hyperarousal *(7 items)* Cronbach’s α = 0.87	- *I feel irritable and angry* - *I am jumpy and easily startled* - *I find myself acting or feeling like I am back at that time*	

To score individuals using the scale, the sum of all 22 items is calculated and reflects an IES-R score, which indicates a level of PTSD symptomology. The original IES-R response scale is a 5-point scale ranging from 0 to 4, with an original score range of 0–88. However, for this study, the response scale was relabelled as 1–5 to remain consistent with the other items in the survey; this has no bearing on the interpretation of scores, other than adjusting the range of possible scores to 22–110 for this study. Subsequent references to IES-R scores in the Results account for this adjustment. When interpreting IES-R scores, studies have typically focused on population-specific explanations which makes it difficult to standardise cutoff scores. However, most studies and clinical references agree that IES-R scores over 26 (which equates scores > 48 in this study) are meaningful and signify that PTSD is a clinical concern^60,61^. According to Asukai et al. (2002), those with scores this high who do *not* have full PTSD will have partial PTSD or at least some of the symptoms. A psychometric study of the IES-R by Creamer et al. (2003) found that scores of 33 and above (≥ 55 in the present study) represent the best cutoff for a likely diagnosis of PTSD; and scores of 37 or more (≥ 59 in the present study) can be high enough to suppress the immune system up to 10 years after an impact event^64^. However, as stated earlier, the objective of this study was not to clinically diagnose PTSD but rather use these measurements to describe the psychological experience.

### Data analysis

Data were analysed using STATA/BE 17.0 for Windows^65^. Frequencies, descriptive statistics (e.g. means, standard deviations), correlations, and t-Tests were conducted, followed by a path analysis.

As participants were recruited through two different pathways, namely online research panel and externally shared link (referred to as ‘social media’ link, hereafter), t-Tests were conducted to compare means between panel and social media participants across all variables including age and gender; similar comparisons were also calculated between farmers and non-farmers (see Supplementary Table S2 for all comparisons). This was done to check that the sample groupings did not unduly influence results. Mean comparisons indicated some significant differences across sample type and farmer status on the model variables (see Supplementary Table S2). However, the resultant effect sizes in most cases were considered small or very small based on interpretation of Cohen’s *d* values^66^. Nonetheless, to account for any potential influence that sample type or farmer status may have had on the interpretation of data, both ‘*sample’* and ‘*farmer’* were included as predictor variables in the path model.

In summary, as an exploratory study, a total of 18 predictor variables were included in the path model analysis (including age, gender, sample type and farmer status), along with the two outcome variables. Due to the large number of variables included, stringent significance levels were applied when interpreting the final results (*p* ≤ 0.01) so as to minimise risk of making a type 1 error^67–69^.

## Results

In this section, we present descriptive statistics (i.e. frequencies, means, standard deviations, correlations) for key variables included in our study, as well as reporting on the final path model analysis. Key results are discussed within text; the remainder can be found in Supplementary Tables S1–S2, Supplementary Results, and Supplementary Figure S1.

**Fig. 3:**
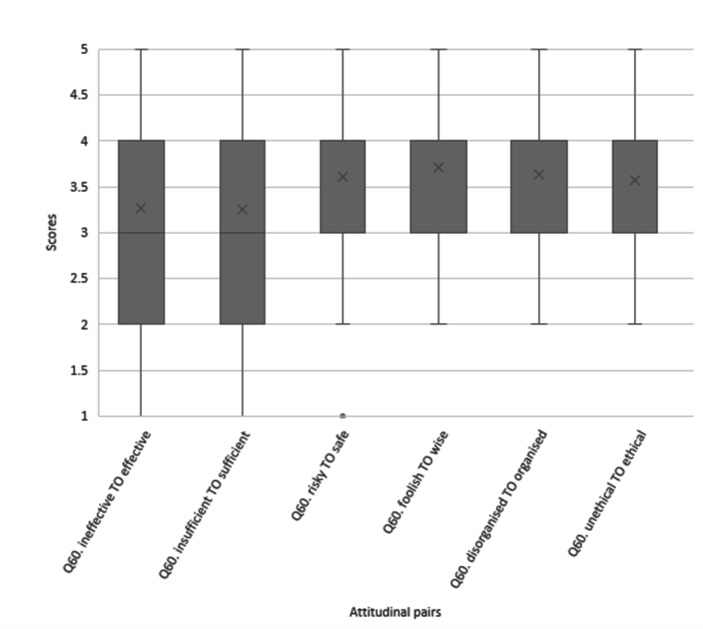
Frequency of scores representing attitudes towards personal responses to the 2021 NSW mouse plague; lower scores represent closer alignment with the negative adjective and higher scores show alignment with the positive adjective.

In total, 1,691 people participated in the online survey; 18% of the sample (*n* = 309) identified as being a *farmer*. Self-reported *attitudes* towards one’s personal response in dealing with the mouse plague were more positive than negative (Fig. 3). However, almost half the total sample (49%) felt as though they were not in *control* of managing mice on their property during the plague. Generally, it was agreed amongst most participants that personal *response costs* associated with controlling mice during the plague involved a lot of personal effort (80%), time (77%) and money (72%). When participants were asked if the impacts of the mouse plague were so bad that they engaged in behaviours they knew were wrong (e.g. knowingly using the wrong bait), 18% reported engaging in *poor behaviours* during the plague while 63% disagreed to the statement. However, one in five respondents chose to ‘neither agree nor disagree’ to the statement, suggesting a high number of neutral (or non-) responses.

Individual perceptions of *animal welfare* during the mouse plague showed only 11% of participants felt the welfare of mice was a major concern for them. A comparison of means showed that women perceived slightly stronger animal welfare concerns during the mouse plague than men (t_917.40_ = -6.20, *p* < 0.001, Cohen’s d = 0.33). Despite the overwhelming lack of concern for mouse welfare, almost 30% of the sample reported feeling sad when killing mice.

### Support during the plague

During the mouse plague, most participants (64%) felt they could count on the *social support* of 1–2 others for help and 41% believed others held moderate concern for them (see Table 4 for mean scores). However, almost a quarter (26%) believed that that they could not count on anyone else to help them and 44% believed that people showed little to no interest or concern for them during the plague. Further, respondents believed it was possible (45%) but difficult (37%) to receive practical help from neighbours if they needed it. Interestingly, males perceived a greater level of social support than females during the plague (t_1671_ = 2.30, *p* = 0.022, Cohen’s d = 0.13), though this was associated with a very small effect size. When asked to reflect on aspects of authority (government) support during the mouse plague, average scores fell below the mid-point (*M* = 2.36, *SD* = 1.03; Table 4), indicating lower levels of satisfaction overall. A more detailed presentation of *authority support* can be found in *Supplementary Results*, including thematic analysis of open text questions regarding the perceived role of Government during the 2021 NSW mouse plague (Supplementary Figure S1).

**Table 4:** Mean, standard deviations and correlation matrix for linear variables in the path model (*N* = 1691).

Variable	Mean	SD	Correlation matrix																
			1	2	3	4	5	6	7	8	9	10	11	12	13	14	15	16	17
1. Severity of impacts	13.27	3.27	1	0.43^**^	-0.01	0.03	0.50^**^	0.58^**^	0.31^**^	-0.16^**^	0.68^**^	-0.36^**^	-0.07^**^	0.30^**^	0.07^**^	-0.19^**^	-0.29^**^	0.20^**^	0.46^**^
2. Ongoing psychological stress	38.60	15.63		1	-0.16^**^	0.26^**^	0.62^**^	0.32^**^	0.36^**^	-0.22^**^	0.36^**^	-0.22^**^	0.17^**^	0.33^**^	-0.03	-0.08^**^	-0.16^**^	0.05^*^	0.50^**^
3. Age	48.70	15.64			1	-0.24^**^	-0.21^**^	-0.09^**^	-0.24^**^	0.07^**^	-0.07^**^	0.03	-0.22^**^	-0.17^**^	-0.06^*^	0.02	0.05	0.09^**^	-0.19^**^
4. Neuroticism	22.52	6.03				1	0.43^**^	0.11^**^	0.23^**^	-0.17^**^	0.02	-0.09^**^	0.13^**^	0.10^**^	-0.09^**^	0.01	-0.01	-0.02	0.17^**^
5. Depression	20.21	7.37					1	0.41^**^	0.42^**^	-0.30^**^	0.46^**^	-0.35^**^	0.10^**^	0.31^**^	-0.09^**^	-0.18^**^	-0.26^**^	0.10^**^	0.49^**^
6. Disgust-smell	3.96	1.07						1	0.34^**^	-0.19^**^	0.57^**^	-0.34^**^	-0.04	0.27^**^	-0.01	-0.18^**^	-0.24^**^	0.22^**^	0.38^**^
7. Embarrassment	3.66	1.20							1	-0.25^**^	0.35^**^	-0.29^**^	0.16^**^	0.26^**^	-0.12^**^	-0.13^**^	-0.20^**^	0.10^**^	0.39^**^
8. Attitudes	3.51	0.71								1	-0.15^**^	0.37^**^	-0.15^**^	-0.33^**^	0.12^**^	0.12^**^	0.17^**^	− 0.001	-0.23^**^
9. Response costs	11.82	2.46									1	-0.39^**^	-0.06^*^	0.26^**^	-0.03	-0.21^**^	-0.33^**^	0.23^**^	0.44^**^
10. Perceived control	2.68	1.10										1	0.05	-0.18^**^	0.09^**^	0.25^**^	0.28^**^	-0.21^**^	-0.29^**^
11. Animal welfare	2.12	0.80											1	0.18^**^	-0.04	0.09^**^	0.06^*^	-0.09^**^	0.05
12. Poor behaviours	2.35	1.15												1	0.02	-0.02	-0.09^**^	-0.00	0.33^**^
13. Social support	7.09	2.15													1	0.20^**^	0.24^**^	-0.04	-0.02
14. Communication from authorities	2.49	1.01														1	0.72^**^	-0.13^**^	-0.17^**^
15. Authority support	2.36	1.03															1	-0.13^**^	-0.24^**^
16. Future threat vulnerability	4.35	0.74																1	0.24^**^
17. Future threat severity	3.16	1.05																	1

** Correlation is significant at the 0.001 level * Correlation is significant at the 0.05 level.

#### Psychological traits and emotional states

**Fig. 4:**
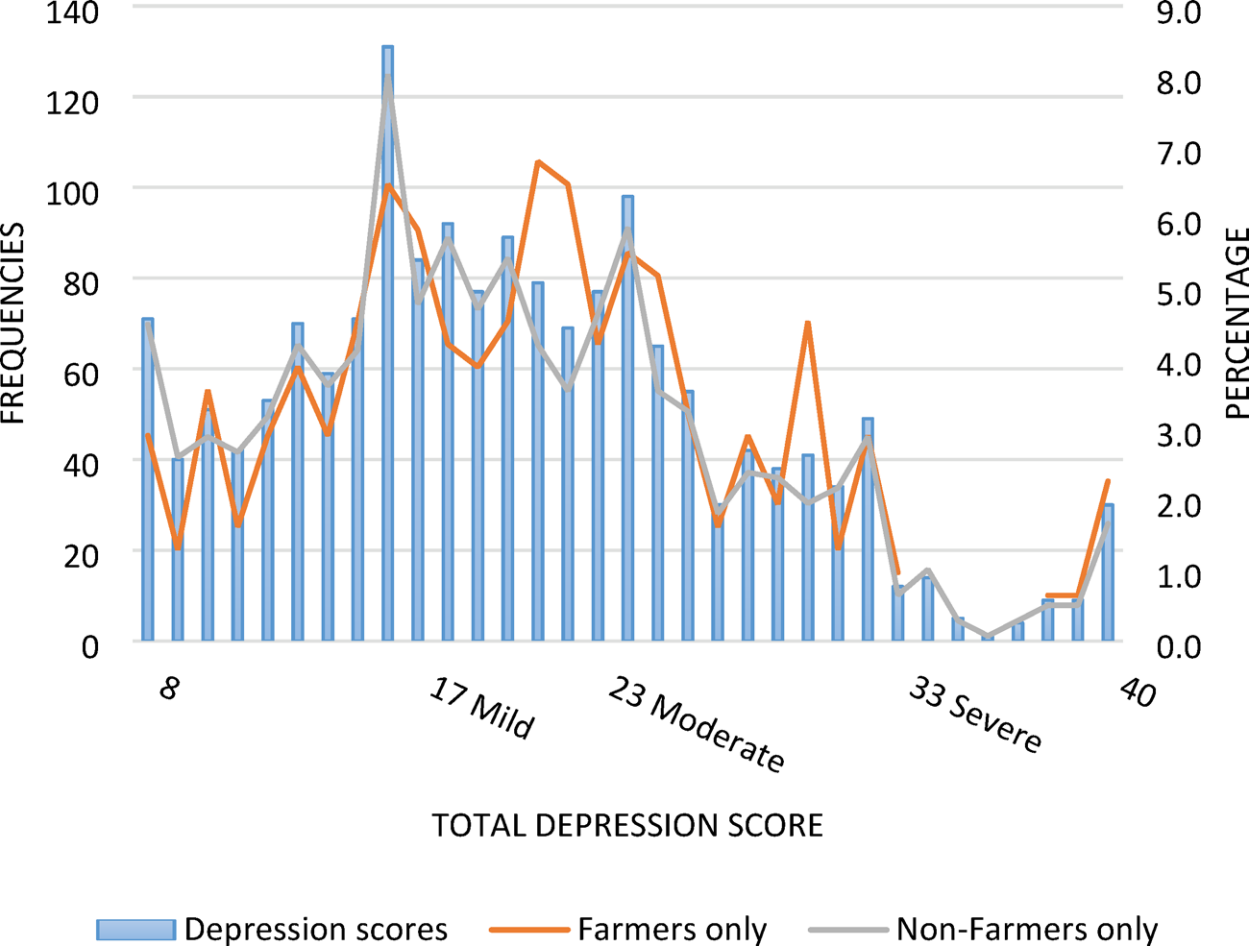
Frequencies of retrospective emotional disturbance (depression) scores for the sample population during the 2021 NSW mouse plague, overlaid with a comparison of scores between farmers and non-farmers. Higher scores indicate more severe depressive symptoms; key interpretation points are signposted along the x-axis^46–48,73^.

A state-based depression scale asked participants to reflect on how they felt during the 2021 plague. Scores ranged from 8 (lowest score possible) to 40 (highest score possible). Participants reported an average emotional disturbance (*depression*) score of 20 (see Table 4), which indicated mild depressive symptoms during the mouse plague^70^. However, further examination of frequency distributions showed that while approximately 35% of the sample reported ‘none to slight’ depressive symptoms, 29% reported mild symptoms, 31% reported moderate symptoms, and approximately 5% of the sample (*n* = 84) reported severe depressive symptoms during the mouse plague (Fig. 4). This general pattern of results did not differ between those who identified as farmers or non-farmers. Note: The measure of *depression* used in this study is not intended as a diagnosis but rather used to illustrate the relative (retrospective) mood disturbance among participants during the 2021 NSW mouse plague. For comparison, according to the latest data release in 2017-18, approximately 9.8% of New South Wales residents had depression or experienced feelings of depression^71^.

Emotive responses to *disgust* at the time of the plague were also measured, as it was thought that disgust associated with the unpleasant odour of mice during the plague might further influence mood^40^. Approximately 42% of people reported being extremely bothered by the odour of mice during the plague and almost two-thirds (63%) of respondents reported feeling *embarrassed* about having mice in their home or business. Disgust and embarrassment had moderate-strong correlations with perceived severity of impacts, and embarrassment was moderately correlated with the experience of depressive symptoms (Table 4).

A measure of the personality trait, *neuroticism*, was also included in the survey to assess whether a trait-based psychological characteristic could exert influence on one’s experience of severity of impacts and ongoing psychological stress. Neuroticism has been described as an underlying personality characteristic influencing how sensitive, emotional, and prone to worry an individual may be^27,72^. Results from the current sample showed that average scores for neuroticism fell within the ‘low’ range (M = 22.52, SD = 6.03), indicating that participants within the sample were not particularly anxious, sensitive, or emotional by nature. This suggests that any negative psychological experiences participants reported relevant to the mouse plague should not be interpreted as being strongly driven by a neurotic personality characteristic.

#### Future-oriented perceptions

Finally, participants were asked how likely it was that they would get mice on their property in the future (*threat vulnerability*) and whether a future mouse plague would be detrimental to them (*threat severity*). Approximately 90% of participants agreed or strongly agreed that it was likely that they would get mice on their property if another mouse plague happened; just over one-third (38%) of participants worried that a future mouse plague would be dangerous or catastrophic to them.

#### Outcome variables: severity of impacts and ongoing psychological stress

Overall, approximately 95% of participants reported at least moderately severe mouse plague impacts on their *community* and 80% reported at least moderately severe personal impacts. Post hoc analyses showed that males experienced significantly less severe impacts than females (t_1671_ = -5.45, *p*<0.001, Cohen’s d = 0.29), with a small-moderate effect size calculated. A comparison was also carried out between farmers and non-farmers and while there was a significant difference in severity of impacts between these two groups, the effect size was considered small (t_491.58_ = 3.51, *p*<0.001, Cohen’s d = 0.21).

**Fig. 5:**
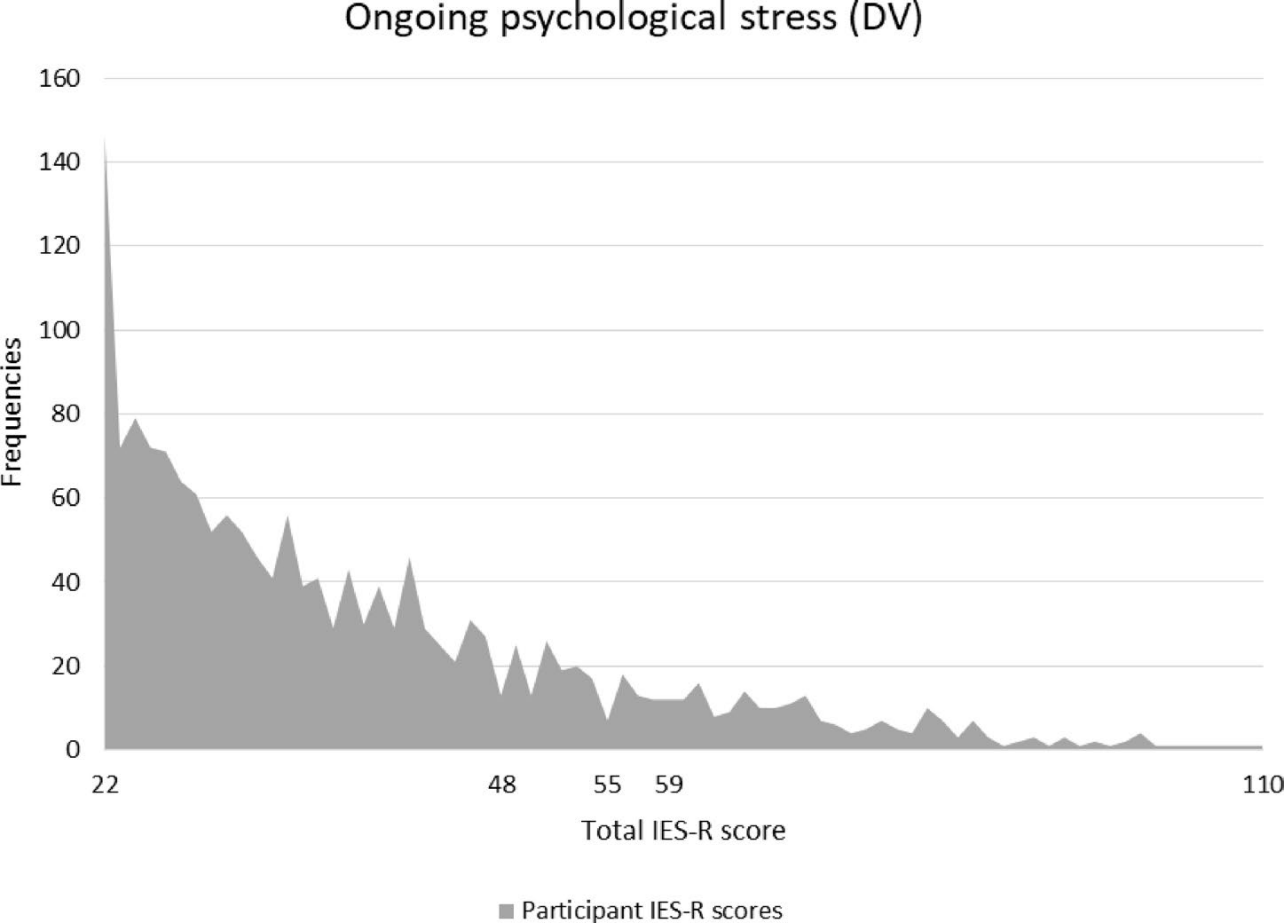
Participant scores for ongoing psychological stress measured two years post-plague; higher scores indicate stronger PTSD symptoms. Score markers on the Y-axis signifying meaningful cut-off points; scores > 48 suggest that PTSD may be a clinical concern.

Ongoing psychological stress amongst participants showed that, on average, participants experienced little or no ongoing psychological stress after the 2021 mouse plague (M = 38.60, SD = 15.63). However, as with depression, further examination of total score frequencies provided a more nuanced picture of ongoing stress (Fig. 5). It was found that 23% of participants scored > 48, indicating that almost one quarter of the sample reported scores that the literature suggests is meaningful and signifies that PTSD may be a clinical concern. That is, individuals with this score and higher are likely to have partial PTSD or at least some of the symptoms. The literature also suggests that scores ≥ 55 represents the best cutoff for a likely diagnosis of PTSD and 15% of the sample scored within this range. Furthermore, 13% of the sampled scored ≥ 59, which is considered severe enough to have implications on immune system suppression. Therefore, although the total mean score for the sample suggests no PTSD concerns, the frequency distributions suggests that almost one in four people may be experiencing at least some symptoms of PTSD.

Gender comparisons showed that the two main groups, males and females, differed in their ongoing psychological stress after the mouse plague, with females reporting higher scores than males (t_1671_ = -3.74, *p*<0.001, Cohen’s *d* = 0.20), but the effect size was considered small. Similarly, females also reported significantly higher depression scores than males (t_1671_ = -6.14, *p*<0.001, Cohen’s d = 0.35), and this showed a more moderate effect size. The non-binary/unstated gender group was too small to conduct valid comparisons on any of the outcome variables.

### Path model

Path analyses were conducted to examine the relationship between key social and psychological (independent) variables and the two dependent (outcome) variables: perceived *severity of social impacts* from the mouse plague, and *ongoing psychological stress* approximately two years after the mouse plague.

The hypothesised fully mediated path model, describing all independent variables predicting *ongoing psychological stress* via *severity of impacts*, was not a good fit for the data. Post hoc model modifications were performed to try and develop a better fitting model. On the basis of modification indices (MIs) and theoretical relevance, direct paths between several of the independent variables and ongoing psychological stress were considered. The largest changes in modification indices through the inclusion of these additional direct relationships were for emotional disturbance (*depression*; MI = 339.67), *neuroticism* (MI = 87.41), perceptions of animal *welfare* (MI = 61.46), and social *embarrassment* (MI = 54.40). Several other direct relationships were also recommended and among those, only the most theoretically meaningful direct relationships were included: knowingly engaging in *poor behaviours* (MI = 41.51), *sample* type (MI = 38.17), *attitudes* towards one’s own response (MI = 17.18), and the demographic variables of *age* (MI = 11.85) and being a *farmer* (MI = 10.37). The model was significantly improved with the addition of these paths, and the final model fit the data well (Table 5).

**Fig. 6:**
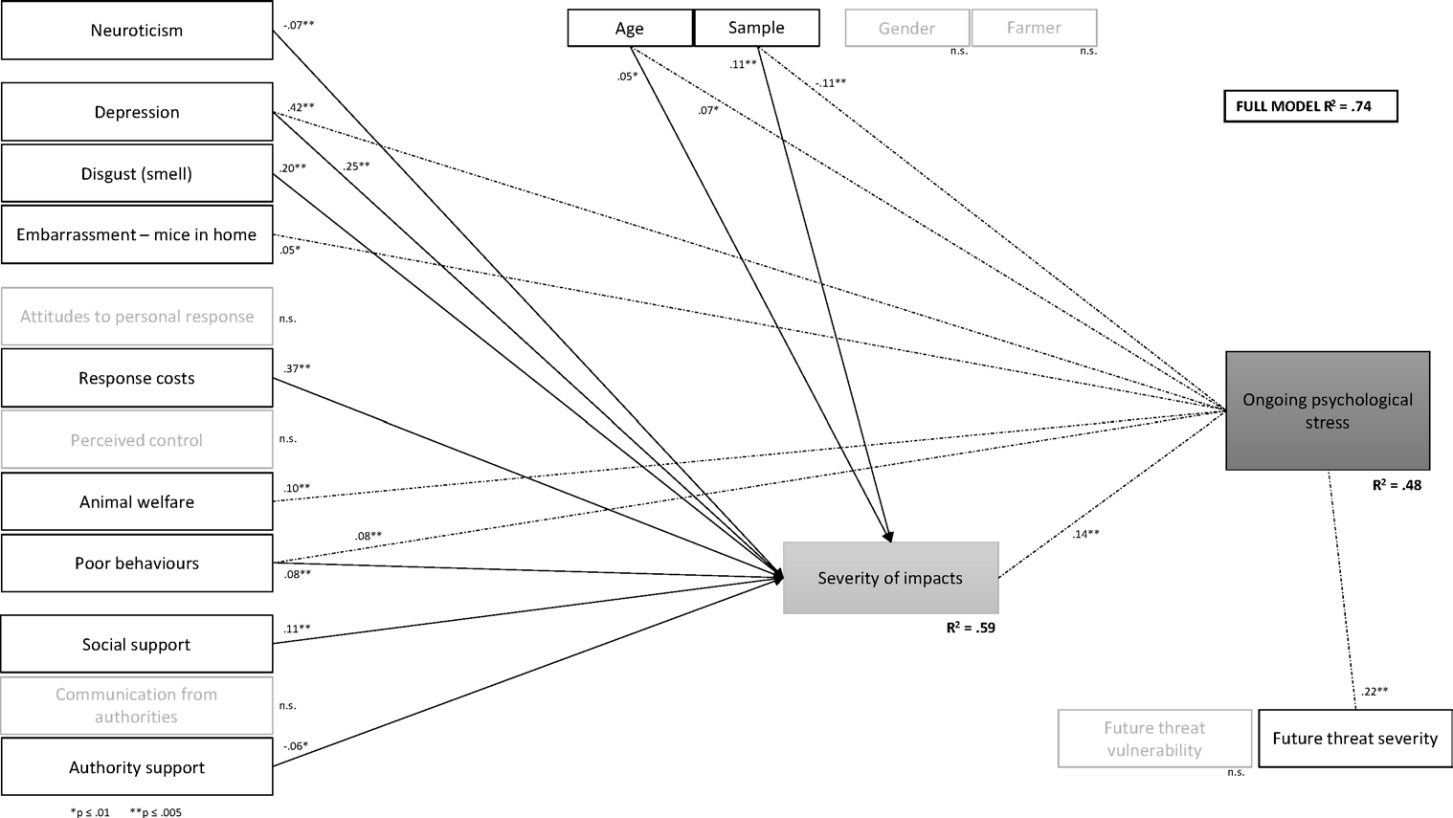
A partially mediated model of psychological, social and demographic factors predicting severity of social impacts and ongoing psychological stress from the 2021 NSW mouse plague. All coefficients shown are significant at *p* < 0.001 unless specified otherwise.

Overall, the predictor (input) variables accounted for 74% of the variability observed in perceived *severity of impacts* and *ongoing psychological stress*. Most associations amongst the variables were in the hypothesised direction and the final partially mediated model with standardised coefficients is presented in Fig. 6. The direct effects model for perceived *severity of impacts* from the 2021 mouse plague was very strong, with the input variables explaining 59% of the variance in *severity of impacts*. *Severity of impacts* was predominantly influenced by perceived *response costs*; that is, the assessment that controlling mice during the plague required a lot of personal effort, time and money, and this was positively associated with increased social impacts. Experiencing negative mood states (i.e. *depression*) was also a strong driver of perceived impact, as well as *disgust* elicited by the odour of mice. Interestingly, *social support* was a significant influence on the severity of social impacts but exerted a weaker influence than other factors and not in the hypothesised direction.

**Table 5:** Fit statistics for the hypothesised (fully mediated) model, compared to the final (partially-mediated) model.

Model	Chi-square (df)	Comparative fit index^§^ (CFI)	Tucker-Lewis index^+^ (TLI)	Standardized root mean Square residual^2^ (SRMR)	Root mean square error of approximation^1^ (RMSEA)	*R*^2^
Recommended good model fit ranges	n.s. [p < 0.05]	≥ 0.95	> 0.90	< 0.05	< 0.05	> 0.50 is a strong model
Hypothesized model (full mediation)	499.44 (18), p < 0.001	0.81	0.61	0.033	0.13	0.65
Final model (partial mediation)	31.06, p = 0.0003	0.99	0.97	0.006	0.04	0.74

^§^^74^^+^^75^.

The direct effects model for *ongoing psychological stress* was also found to be very strong, with the independent variables explaining almost half (48%) of the variance in *ongoing psychological stress* after the mouse plague. The most influential direct predictor of *ongoing psychological stress* was the experience of *depression* or depressive symptoms at the time of the plague, and concerns that a future mouse plague would be dangerous or catastrophic to oneself. *Severity of impacts*, as the mediating variable, was the third-most influential factor (Table 6).

Amongst the demographic factors of age, gender, farmer status and sample type, the most influential variable was *sample* type. While not a strong predictor, *sample* did exert a similar magnitude of influence in the prediction of *severity of impacts* as well as *ongoing psychological stress*. Specifically, participants sourced from social media tended to report experiencing greater *severity of impacts* while the panel sample reported more *ongoing psychological stress* after the plague. *Age* was the only other demographic factor to significantly predict the outcome variables, though the influence for both outcome variables was very weak; older age was associated with greater perceived impacts and higher ongoing psychological stress.

**Table 6:** Standardised coefficients (β) and significance levels (p) for relationships within the final partially mediated path model, using outcome variables of severity of social impacts and ongoing psychological stress.

Dependent (outcome) variable Predictor variables	β	*p*	Mediators Dependent (outcome) variable Predictor variables	β	*p*
Ongoing psychological stress			Severity of impacts		
Depression	0.42	< 0.000	Response costs	0.37	< 0.000
Future severity	0.22	< 0.000	Depression	0.25	< 0.000
Severity of impacts	0.14	< 0.000	Disgust (smell)	0.20	< 0.000
Sample type	-0.11	< 0.000	Social support	0.11	< 0.000
Animal welfare	0.10	< 0.000	Sample type	0.11	< 0.000
Poor behaviours	0.08	< 0.000	Poor behaviours	0.08	< 0.000
Age	0.07	0.001	Neuroticism	-0.07	< 0.000
Embarrassment	0.05	0.010	Age	0.05	0.002
*Future vulnerability*		*n.s.*	Authority support	-0.06	0.009
*Farmer*		*n.s.*	*Animal welfare*		*n.s.*
*Attitudes to personal response*		*n.s.*	*Attitudes to personal response*		*n.s.*
*Neuroticism*		*n.s.*	*Perceived control*		*n.s.*
			*Communication from authorities*		*n.s.*
			*Farmer*		*n.s.*
			*Embarrassment*		*n.s.*
			*Gender*		*n.s.*
n.s. *is non-significant at p > 0.01*					

## Discussion

This study is the first to describe and quantify the psychosocial dimensions of a pest incursion, using the case of the 2021 NSW mouse plague to model social impacts and ongoing psychological burden. Social impacts are rarely measured or modelled; yet these factors are critically important in developing a nuanced understanding of the effect that severe pest invasions can have on people in affected communities. Livelihoods in these communities often depend on the agricultural sector and mental wellbeing can be persistently affected after such an event. Results showed that demographic characteristics such as age and gender did not matter significantly in relation to experiencing the intensity of social impacts of the plague; and farmers and non-farmers alike experienced psychosocial effects similarly, both at the time of the plague and in the years following the event. Further, past personal experience of a mouse plague did not necessarily prepare individuals to better cope with the 2021 plague as the literature may have predicted.

What seems to be most important in the intensity of lived social impacts during the plague was the overwhelming feeling that dealing with the plague cost oneself large amounts of money, time and effort. This concept of “response cost” can be defined as the toll taken to respond adaptively to a threat and reflects an evaluation of one’s coping resources. The literature suggests that higher perceived response costs can influence one’s coping behaviour maladaptively^76^; consequently, those with higher perceived response costs may be less likely to respond adaptively or functionally to a threat event. The significant correlation between response costs and depressive symptoms in this study lends support to this hypothesis.

High emotional disturbance (depression) was prevalent amongst a significant proportion of the surveyed population. People who regularly felt sad, fearful, worried, angry or helpless during the height of the plague were more likely to experience negative changes to their sleep, eating and social habits, which may have compounded their perceptions of negative social impacts. These emotional responses align with core features of psychological trauma, which suggests that exposure to an uncontrollable, threatening event can lead to such intense stress responses which can result in lasting psychological harm^18,19,77^. In this context, the mouse plague can be conceptualised as a traumatic stressor, with depressive symptomology acting not only as a marker of distress, but also as an indicator of longer-term psychological outcomes. This was evidenced by the strong relationship between depression and PTSD symptoms amongst participants who reported ongoing psychological stress. Experiencing depression during the event may have impaired coping mechanisms or heightened sensitivity to subsequent stressors post-plague, thereby increasing vulnerability to trauma-related impacts^30^.

Importantly, emotional disturbance and ongoing stress were only very weakly associated with preexisting characteristics of neuroticism, lending support to the interpretation that retrospective depression scores and ongoing stress post-plague were more likely attributable to the mouse plague event rather than underlying emotional instability. This pattern is consistent with findings from other disaster contexts – such as bushfires and floods – where acute environmental stressors have been shown to trigger trauma-like responses in otherwise psychologically stable populations^27,56^. What did seem to influence ongoing psychological stress, aside from depression during the plague, was the worry that a future mouse plague would be personally dangerous or catastrophic. Emerging literature on the relationship between posttraumatic stress symptoms and threat unpredictability^78–80^suggests that not only can PTSD cause a heightened reaction to a threat, but also that unpredictable threats such as a mouse plague – or indeed any other extreme pest disaster – can cause more severe PTSD symptoms when compared to the effects of a predictable threat (e.g. seasonal weather patterns). This further suggests that the mouse plague has both acute and chronic effects on individuals.

Another key traumatic influence on one’s experience of the plague was the negative psychological/emotional influence of odour and its high correlation with perceived response costs, as well as its role as an influential predictor of higher negative social impacts^40^. Carter and colleagues^40^also described the overwhelming effect of putrid odour of dead and dying mice on one’s increased psychological burden during the 2021 plague. While they found qualitative evidence to suggest that odour was likely an emotional memory trigger for some, the quantitative modelling in this study showed that the influence of odour on psychological wellbeing seemed limited to the plague’s duration and did not directly influence ongoing psychological stress. Our results showed that people felt a high amount of psychological discomfort associated with having mice present in their homes and/or businesses, and this contributed to the experience of co-occurring negative emotions such as anxiety, guilt, shame.

In addition to the myriad stressors individuals experienced as a result of the mouse plague, it is important to consider that there may be a broader spectrum of stress responses that environmental disruptions can elicit, such as physiological and other psychological-based responses, that have not been measured in the study^32,64,81,82^. By design, this study narrows its scope to focus on two dominant experiences – namely depression and post-traumatic stress – based on recent literature exploring the social experience of the mouse plague^40^. However, there are many other facets of the human stress response that may also be playing a role (e.g. cultural norms, learned behaviours, chronic stressors, preexisting psychological conditions, past traumas), which were measured in this study, providing a rich area for future research.

Further, the potential for comorbid psychological effects arising from other significant regional stressors – both leading up to the 2021 mouse plague and in the two years that followed. In particular, the region was affected by prolonged drought conditions and these overlapping environmental challenges may have compounded the emotional burden experienced by individuals, either by intensifying the perceived severity of social impacts from the plague, or by reducing psychological resilience due to cumulative stress exposure^30,81,83^. Future research could benefit from disentangling the specific and interactive effects of concurrent disasters to better understand the full scope of trauma-related outcomes in affected communities.

While the physical health advice for mouse plagues provided by State and Federal governments continues to focus on prevention of infectious disease, the broader dimensions of wellbeing examined in this study, such as the mental health burden of mouse plagues, have not yet been discussed widely. There is currently no tailored mental health support to address the specific experiences of people suffering mouse plagues as a disastrous event. For example, the *Staying Healthy During a Mouse Plague* factsheet published by NSW government does not mention mental health as a concern in its public health advice^84^. The Australian Government Department of Agriculture, Fisheries and Forestry does list the contact details of general mental health services on their public advice page for dealing with mice, but these are not specifically tailored for experiencing plague events^85^. While local wellbeing programs (e.g. Rural Adversity Mental Health Program in NSW)^86^offer services in some plague-affected areas, along with the support provided by local charities like The Salvation Army Australia, provision of dedicated mental health advice could be a possible policy consideration in preparation for the next plague event. Health advice could cover aspects of wellbeing not directly related to zoonoses and might include mental and emotional health, along with other physical health related dimensions of wellbeing like sleep disturbance and fatigue management, as experienced in similar international contexts^87,88^.

Perceptions of social support, both from friends and family as well as relevant authorities, were measured in this study and showed interesting relationships. Individual social support levels were generally reported as being low among the surveyed population, and the direction of the relationship between social support and perceived severity of impacts suggested that the more social support one received, the higher their reported social impacts. This suggests that receiving help and care from others may have had a slightly adverse effect on one’s experience of the mouse plague. A possible explanation for this could be that the sharing of others’ trauma or negative experiences burdened individuals such that it negatively affected their own perceptions of the plague^40^. Indeed Caughley’s^8^work during the 1993 mouse plague found a similar absence of instrumental social support, namely due to a high level of internalised stress amongst affected individuals, and a general lack of community coherence that might be expected during a natural disaster. The broader stress literature typically shows that increased social support during disaster events can protect individuals against depression and PTSD^34,36^; however, there is evidence in the disaster literature that not all social support is received positively, and it can depend on who is providing the support and whether support is perceived as benevolent or not^89^. A review by Kaniasty^90^found inconsistent evidence that social support positively influenced survivors’ wellbeing, and that in some instances, availability of social support could be restricted to in-groups at the exclusion of others.

The results highlight the complexity in the collective experience of the plague, where individuals are trying to manage their own experience, while also trying to prioritise the needs of others in the community^90^. It is also clear that community dynamics warrant greater consideration when designing social supports in preparation for future plague events. Well-targeted future research to support mental and community health outcomes during and after plague might focus on improving the purpose and timing of support provision. Drawing on existing frameworks and research on maintaining mental health during natural disasters could be incorporated as part of disaster planning^81,91^.

### Conclusions and limitations

Results from this study demonstrate that NSW regional communities experienced significant social impacts during the 2021 mouse plague and provides compelling evidence for the trauma hypothesis after a severe pest incursion. It was found that approximately one in three survey participants reported depressive symptoms during the plague and one in four respondents continued to experience ongoing PTSD-like symptoms two years post-plague. To date there does not appear to be much – if any – literature available measuring these types of impacts in relation to a severe pest incursion. This often-overlooked aspect of agricultural disasters can have a considerable impact on the health of the broader community.

Whilst this study looked to capture a range of social and psychological influences on one’s experience of the mouse plague, it should be noted that the large number of variables within the model makes it more susceptible to issues such as overfitting, multicollinearity, and complex model interpretation. To mitigate these risks, a more conservative *p* threshold was used, and interpretations were based on effect sizes; however, some relationships may not have been clear. Also, as with all studies utilising self-reported data, it is cautioned that results may be subject to bias. Additionally, this study utilised two recruitment pathways for the sample, to enable a more inclusive recruitment process, while still also retaining a portion of the sample that was representative of the demographic profile for the target region. The relative importance of sample type in the path model suggests that while panel recruitment is an excellent way to ensure sample stratification with key demographic indicators within the target region, such as age and gender, it is important that future studies include random sampling from alternative diverse sources such as social media and industry-specific communities.

Overall, these results taken with other emerging literature on the 2021 mouse plague, paint a picture of a disaster event characterised by economic losses, regional and societal disruptions, and individual health impacts. Just as plague impacts are complex and multidimensional, initiatives designed to manage future mouse plague events should reflect these complexities. It is recommended that community leaders, mental health professionals and government authorities work together to develop ways to support at-risk communities, considering interventions that alleviate both acute social impacts *during* a plague as well as longer term psychological impacts *after* a plague. Of particular relevance to these findings is the need for better coordinated responses across various government departments, research and civil society sectors to ensure all aspects of human wellbeing are considered during and following plague events^7^. Acknowledging that plague events expose risks to individuals, families and communities, and that these risks extend beyond the farm gate is a first crucial step. Pest incursions can no longer be treated as simply biosecurity or agricultural problems alone – these events affect specific dimensions of human wellbeing, none of which are presently adequately addressed through existing programs.

## Supplementary Information

Below is the link to the electronic supplementary material.

Supplementary Material 1

Supplementary Material 2

Supplementary Material 3

Supplementary Material 4

## Data Availability

The dataset generated and analysed within the current study is available from the corresponding author on reasonable request.
