# Supplementary material for: Psychosocial impacts of a mouse plague and ongoing psychological stress
Source: Sci Rep. 2026 Feb 11;16:8390. doi: 10.1038/s41598-026-39861-1 (PMC12972104; doi:10.1038/s41598-026-39861-1)
Supplement: Supplementary file 1 — Supplementary Material 1 [file 41598_2026_39861_MOESM1_ESM.docx]

**TITLE:** Psychosocial impacts of a mouse plague and ongoing psychological stress

**AUTHORS**: Aditi Mankad*, Kerry Collins, Walter Okelo, Lucy Carter & Peter Brown

***CORRESPONDING AUTHOR DETAILS:** Aditi Mankad, CSIRO Environment, GPO Box 2583, Brisbane QLD 4001, AUSTRALIA; [aditi.mankad@csiro.au](mailto:aditi.mankad@csiro.au)

**Supplementary Results: Authority (government) support**

This supplementary material is references in *Section 3. Results*, specifically relating to *Section 3.1 Support during the plague*. It provides further information on how people perceived authority support during the 2021 mouse plague, as well as public expectations regarding authority support during this time.

**Results summary**

When asked to reflect on aspects of authority (government) support during the mouse plague, average scores fell below the mid-point (*M* = 2.36, *SD* = 1.03; Table 5), indicating lower levels of satisfaction overall. Frequencies showed that most people disagreed (32%) or strongly disagreed (24%) when asked if they were satisfied with the level of support provided by government. A smaller proportion agreed (13%) or strongly agreed (2%) that they were satisfied with the level of authority support provided. Further, 51% of people disagreed or strongly disagreed the government was proactive in informing them about the 2021 mouse plague. These findings were consistent across both farmer and non-farmer subsamples (t_1689_ = -0.67, *n.s.*); however, panel participants were more satisfied with government support than social media respondents (t_1689_ = 8.02, p<.001, Cohen’s d = 0.41).

To contextualise perceptions of government support, participants were asked to articulate what they believed the role of the government authority was during the 2021 mouse plague. Participants predominantly reported that government had a role in providing the impacted community with social support; communication and education on issues related to managing the mouse plague; and, financial support (e.g. rebates). Other key themes included government’s role in direct mouse control; providing residents with pest control resources (i.e., baits and traps); regulating mouse control aids (i.e., ensuring supply, approval of control methods, and monitoring cost of baits/traps); and, being prepared for, and responding quickly to, future plague events. Minor themes included an enabling role for government in providing health services (including mental health services); and funding research into improved control methods. Figure S1 shows the themes identified by participants, with circle size representing relative frequency of citation.
