# Supplementary material for: Psychosocial impacts of a mouse plague and ongoing psychological stress
Source: Sci Rep. 2026 Feb 11;16:8390. doi: 10.1038/s41598-026-39861-1 (PMC12972104; doi:10.1038/s41598-026-39861-1)
Supplement: Supplementary file 2 — Supplementary Material 2 [file 41598_2026_39861_MOESM2_ESM.docx]

**TITLE:** Psychosocial impacts of a mouse plague and ongoing psychological stress

**AUTHORS**: Aditi Mankad*, Kerry Collins, Walter Okelo, Lucy Carter & Peter Brown

***CORRESPONDING AUTHOR DETAILS:** Aditi Mankad, CSIRO Environment, GPO Box 2583, Brisbane QLD 4001, AUSTRALIA; [aditi.mankad@csiro.au](mailto:aditi.mankad@csiro.au)

**SUPPLEMENTARY MATERIAL 2: Mean comparisons**

This supplementary material presents t-Tests that were conducted to compare means between a) panel and social media participants, and b) farmers and non-farmers, across all variables including age and gender, as references in Section *2.3 Data analysis* and *3. Results*.

**Summary Results**

It was found that the means between the panel and social media samples significantly differed across most of the variables examined; however, the resultant effect sizes were predominantly small based on interpretation of Cohen’s *d* values (Cohen, 1988). It was noted that the difference between means for the dependent variable *severity of impacts* did have a large effect size; therefore, although the two sample types were combined for subsequent statistical analyses, it was decided that ‘*sample’* would be included as a predictor variable in the path model. This was to account for any influence sample type may have had on the interpretation of data. There was no significant difference between sample type for the second outcome variable of *ongoing psychological stress*.

Preliminary analyses also indicated that there were significant differences between farmers and non-farmers on some variables, including *severity of impacts*. However, all significant differences between farmers and non-farmers were found to have small or very small effect sizes; there was no significant difference found for *ongoing psychological stress*. Even so, the decision was again made to include ‘*farmer*’ as a predictor in the path model, to account for any potential influences of this demographic characteristic on the outcome variables.

Table S2 Comparison of means across all variables used in the path model, between the panel and social media sub-sample, and farmer/non-farmer sub-sample, using t-Tests and Cohen's d for effect size.

| **Variable** | **Sample** | **N** | **Mean** | **SD** | **t^#^** | **df** | ***p*** | **Cohen’s *d*** |
| --- | --- | --- | --- | --- | --- | --- | --- | --- |
| **DV: Severity of impacts** | Panel | 806 | 12.03 | 3.14 | -15.92 | 1689 | .00 | -0.78 |
|  | Social media | 885 | 14.39 | 2.97 |  |  |  |  |
|  | Farmer | 309 | 13.82 | 3.00 | 3.51 | 491.58 | .00 | 0.21 |
|  | Non-farmer | 1382 | 13.14 | 3.32 |  |  |  |  |
| **DV: Ongoing psychological stress** | Panel | 806 | 38.38 | 16.76 | -0.54 | 1689 | .59 | -- |
|  | Social media | 885 | 38.79 | 14.55 |  |  |  |  |
|  | Farmer | 309 | 37.74 | 14.51 | -1.13 | 487.03 | .26 | -- |
|  | Non-farmer | 1382 | 38.79 | 15.87 |  |  |  |  |
| **Age** | Panel | 806 | 43.69 | 15.94 | -13.11 | 1603.77 | .00 | -0.64 |
|  | Social media | 885 | 53.26 | 13.88 |  |  |  |  |
|  | Farmer | 309 | 49.80 | 14.47 | 1.45 | 488.44 | .15 | 0.09 |
|  | Non-farmer | 1382 | 48.45 | 15.89 |  |  |  |  |
| **Gender** | Panel | 806 | 1.69 | 0.46 | -2.24 | 1644.82 | .03 | -0.11 |
|  | Social media | 867 | 1.74 | 0.44 |  |  |  |  |
|  | Farmer | 307 | 1.67 | 0.47 | -2.13 | 435.95 | .03 | -0.14 |
|  | Non-farmer | 1366 | 1.73 | 0.44 |  |  |  |  |
| **Neuroticism** | Panel | 806 | 23.60 | 6.16 | 7.13 | 1643.84 | .00 | 0.35 |
|  | Social media | 885 | 21.53 | 5.73 |  |  |  |  |
|  | Farmer | 309 | 21.85 | 5.51 | -2.30 | 493.91 | .02 | -0.14 |
|  | Non-farmer | 1382 | 22.67 | 6.13 |  |  |  |  |
| **Depression** | Panel | 806 | 19.41 | 7.38 | -4.29 | 1689 | .00 | -0.21 |
|  | Social media | 885 | 20.94 | 7.29 |  |  |  |  |
|  | Farmer | 309 | 20.87 | 7.22 | 1.75 | 1689 | .08 | -- |
|  | Non-farmer | 1382 | 20.06 | 7.39 |  |  |  |  |
| **Disgust-smell** | Panel | 806 | 3.64 | 1.10 | -12.21 | 1601.30 | .00 | -0.60 |
|  | Social media | 885 | 4.25 | 0.96 |  |  |  |  |
|  | Farmer | 309 | 4.12 | 0.98 | 3.08 | 492.06 | .00 | 0.18 |
|  | Non-farmer | 1382 | 3.92 | 1.09 |  |  |  |  |
| **Embarrassment** | Panel | 806 | 3.54 | 1.18 | -3.84 | 1689 | .00 | -0.19 |
|  | Social media | 885 | 3.76 | 1.21 |  |  |  |  |
|  | Farmer | 309 | 3.45 | 1.28 | -3.27 | 433.16 | .00 | -0.22 |
|  | Non-farmer | 1382 | 3.71 | 1.18 |  |  |  |  |
| **Attitudes** | Panel | 806 | 3.56 | 0.72 | 2.99 | 1689.00 | .00 | 0.15 |
|  | Social media | 885 | 3.46 | 0.70 |  |  |  |  |
|  | Farmer | 309 | 3.54 | 0.65 | 0.90 | 1689.00 | .37 | -- |
|  | Non-farmer | 1382 | 3.50 | 0.73 |  |  |  |  |
| **Response costs** | Panel | 806 | 3.64 | 0.85 | -15.32 | 1543.57 | .00 | -0.75 |
|  | Social media | 885 | 4.21 | 0.68 |  |  |  |  |
|  | Farmer | 309 | 4.11 | 0.72 | 4.42 | 511.34 | .00 | 0.25 |
|  | Non-farmer | 1382 | 3.90 | 0.84 |  |  |  |  |
| **Perceived control** | Panel | 806 | 3.01 | 1.01 | 12.41 | 1688.70 | .00 | 0.60 |
|  | Social media | 885 | 2.38 | 1.09 |  |  |  |  |
|  | Farmer | 309 | 2.59 | 1.08 | -1.50 | 1689 | .13 | -- |
|  | Non-farmer | 1382 | 2.70 | 1.10 |  |  |  |  |
| **Animal welfare** | Panel | 806 | 2.22 | 0.82 | 4.56 | 1689 | 0.00 | 0.22 |
|  | Social media | 885 | 2.04 | 0.78 |  |  |  |  |
|  | Farmer | 309 | 1.94 | 0.78 | -4.41 | 1689 | 0.00 | -0.28 |
|  | Non-farmer | 1382 | 2.16 | 0.80 |  |  |  |  |
| **Poor behaviours** | Panel | 806 | 2.30 | 1.08 | -1.85 | 1688.70 | 0.06 | -- |
|  | Social media | 885 | 2.40 | 1.21 |  |  |  |  |
|  | Farmer | 309 | 2.34 | 1.11 | -0.22 | 1689.00 | 0.82 | -- |
|  | Non-farmer | 1382 | 2.36 | 1.16 |  |  |  |  |
| **Social support** | Panel | 806 | 7.25 | 2.12 | 2.84 | 1689 | .00 | 0.14 |
|  | Social media | 885 | 6.95 | 2.16 |  |  |  |  |
|  | Farmer | 309 | 7.37 | 2.12 | 2.51 | 1689 | .01 | 0.16 |
|  | Non-farmer | 1382 | 7.03 | 2.15 |  |  |  |  |
| **Communication from authorities** | Panel | 806 | 2.69 | .97 | 8.02 | 1689 | .00 | 0.39 |
|  | Social media | 885 | 2.30 | 1.01 |  |  |  |  |
|  | Farmer | 309 | 2.45 | 1.04 | -0.68 | 1689 | .50 | -- |
|  | Non-farmer | 1382 | 2.50 | 1.00 |  |  |  |  |
| **Authority support** | Panel | 806 | 2.58 | 1.01 | 8.51 | 1689 | .00 | 0.41 |
|  | Social media | 885 | 2.16 | 1.00 |  |  |  |  |
|  | Farmer | 309 | 2.36 | 1.06 | -0.08 | 1689 | .94 | -- |
|  | Non-farmer | 1382 | 2.36 | 1.02 |  |  |  |  |
| **Future threat vulnerability** | Panel | 806 | 4.14 | 0.76 | -11.24 | 1689 | 0.00 | -0.55 |
|  | Social media | 885 | 4.53 | 0.66 |  |  |  |  |
|  | Farmer | 309 | 4.49 | 0.69 | 3.79 | 1689 | 0.00 | 0.24 |
|  | Non-farmer | 1382 | 4.31 | 0.74 |  |  |  |  |
| **Future threat severity** | Panel | 806 | 3.00 | 1.01 | -5.97 | 1686.38 | 0.00 | -0.29 |
|  | Social media | 885 | 3.31 | 1.07 |  |  |  |  |
|  | Farmer | 309 | 3.30 | 0.99 | 2.57 | 1689 | 0.01 | 0.16 |
|  | Non-farmer | 1382 | 3.13 | 1.07 |  |  |  |  |

^#^ Applying Levene’s Test for Equality of Variances
