# Supplementary material for: Psychosocial impacts of a mouse plague and ongoing psychological stress
Source: Sci Rep. 2026 Feb 11;16:8390. doi: 10.1038/s41598-026-39861-1 (PMC12972104; doi:10.1038/s41598-026-39861-1)
Supplement: Supplementary file 3 — Supplementary Material 3 [file 41598_2026_39861_MOESM3_ESM.docx]

**TITLE:** Psychosocial impacts of a mouse plague and ongoing psychological stress

**AUTHORS**: Aditi Mankad*, Kerry Collins, Walter Okelo, Lucy Carter & Peter Brown

***CORRESPONDING AUTHOR DETAILS:** Aditi Mankad, CSIRO Environment, GPO Box 2583, Brisbane QLD 4001, AUSTRALIA; [aditi.mankad@csiro.au](mailto:aditi.mankad@csiro.au)

**SUPPLEMENTARY Table S1: Sample descriptions**

This supplementary material adds further details regarding the survey sample, as referenced in *Section 2.2.1 Demographic and descriptor variables*.

Table S1 General descriptor variables included in the online survey

| **Variable** | **Example Items** | **Response scale** |
| --- | --- | --- |
| **Years in local area** | How many years have you lived in this NSW regional area? | Open numerical response |
| **Problem normalisation** | I think mouse plagues are a normal part of living in rural areas of Australia | 1 = strongly disagree  3 = neither agree or disagree  5 = strongly agree |
| **Past experience** | *How much experience did you have with a mouse plague, before 2021?* | 1 = no experience at all  5 = extremely experienced |
| **Proportion of household effort** | During the 2021 mouse plague, what proportion of day-to-day household jobs did you do to manage the mouse plague in and around your home/property? | 1 = I didn’t do any of the household jobs  2 = I did one or two of the household jobs  3 = my household equally shared the jobs around the house  4 = I did most of the household jobs  5 = I did all of the household jobs |
| **Future disaster preparedness** | *Experiencing the 2021 mouse plague has made me better prepared for a future mouse plague* | 1 = strongly disagree  3 = neither agree or disagree  5 = strongly agree |

**Results summary**

Approximately 50% of the sample reported having lived in the region for 23 years or more and 43% believed that mouse plagues were a normal part of living in rural Australia (*problem normalisation*). Indeed, only 37% reported having no *past experience* of a mouse plague prior to 2021, whereas 58% reported having at least some experience with a mouse plague before 2021; 5% reported being extremely experienced. Fifty-seven percent of participants agreed or strongly agreed that experiencing the 2021 mouse plague had made them better prepared for a future mouse plague.

When asked the proportion of day-to-day household jobs that individuals engaged in, to manage the plague around their home/property, around 31% reported sharing the load equally with others in the home; 65% reported doing most or all of the household jobs. Interestingly, men and women differed in the proportion of day-to-day household jobs they reported (*t*_779.16_ = -.8.32, p<.001, Cohen’s *d* = 0.46). The resultant moderate effect size suggested that women typically reported doing “most of the household jobs” during the mouse plague, and men were more likely to report “equally shared the jobs around the house”.
