# Supplementary material for: Psychosocial impacts of a mouse plague and ongoing psychological stress
Source: Sci Rep. 2026 Feb 11;16:8390. doi: 10.1038/s41598-026-39861-1 (PMC12972104; doi:10.1038/s41598-026-39861-1)
Supplement: Supplementary file 4 — Supplementary Material 4 [file 41598_2026_39861_MOESM4_ESM.docx]

**TITLE:** Psychosocial impacts of a mouse plague and ongoing psychological stress

**AUTHORS**: Aditi Mankad*, Kerry Collins, Walter Okelo, Lucy Carter & Peter Brown

***CORRESPONDING AUTHOR DETAILS:** Aditi Mankad, CSIRO Environment, GPO Box 2583, Brisbane QLD 4001, AUSTRALIA; [aditi.mankad@csiro.au](mailto:aditi.mankad@csiro.au)


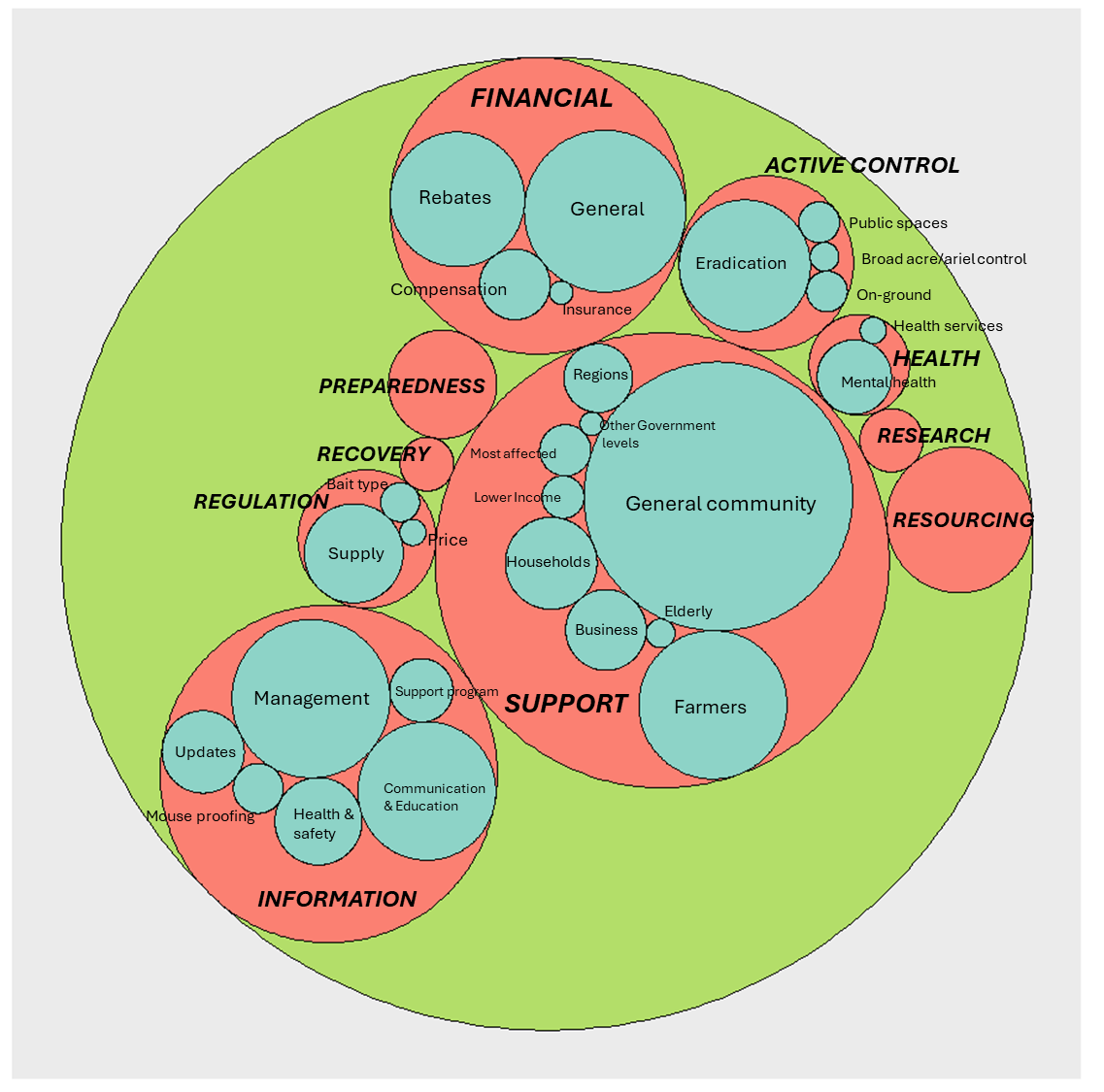


Figure S1 Thematic categories emerging regarding the perceived role of Government during the 2021 NSW mouse plague; larger circles represent stronger themes.
